# Risk assessment in patients with functional class II pulmonary arterial hypertension: Comparison of physician gestalt with ESC/ERS and the REVEAL 2.0 risk score

**DOI:** 10.1371/journal.pone.0241504

**Published:** 2020-11-11

**Authors:** Sandeep Sahay, Adriano R. Tonelli, Mona Selej, Zachary Watson, Raymond L. Benza

**Affiliations:** 1 Houston Methodist Lung Center, Houston Methodist Hospital, Houston, Texas, United States of America; 2 Respiratory Institute, Cleveland Clinic, Cleveland, Ohio, United States of America; 3 Actelion Pharmaceuticals US, Inc., South San Francisco, California, United States of America; 4 Putnam Associates, Boston, Massachusetts, United States of America; 5 Allegheny General Hospital, Pittsburgh, Pennsylvania, United States of America; Vanderbilt University Medical Center, UNITED STATES

## Abstract

**Background:**

Accurate and regular risk assessment is important for evaluation and treatment of pulmonary arterial hypertension (PAH) patients, including those with functional class (FC) II symptoms, a population considered at low risk for disease progression. Risk assessment methods include subjective and objective evaluations. Multiparametric assessments include tools based on the European Society of Cardiology/European Respiratory Society (ESC/ERS) guidelines (COMPERA and FPHR methods, respectively) and the Registry to Evaluate Early and Long-Term PAH Disease Management (REVEAL; REVEAL 2.0 tool). To better understand risk status determination in FC II patients, we compared physician-reported risk assessments with objective multiparameter assessment tools.

**Methods:**

This retrospective chart analysis included PAH patients with FC II symptoms receiving monotherapy or dual therapy. Physicians were surveyed (via telephone) to obtain an assessment of patient risk using their typical methodology, which might have been informed by objective risk assessment. Patient risk was then calculated independently using COMPERA, FPHR and REVEAL 2.0 tools. Factors associated with incongruent risk assessment were identified.

**Results:**

Of the 153 patients, 41%, 46%, and 13% were classified as low, intermediate, and high risk, respectively, by physicians. Concordance between physician gestalt and objective methods ranged from 43%–54%. Among patients considered as low risk by physician gestalt, 4%–28% were categorized as high risk using objective methods. The most common physician factor associated with incongruent risk assessment was less frequent echocardiography during follow-up (every 7–12 months vs. every 3 months; p = 0.01).

**Conclusions:**

More than half of FC II PAH patients were classified as intermediate/high risk using objective multiparameter assessments. Incorporating objective risk-assessment algorithms into clinical practice may better inform risk assessment and treatment strategies.

## Introduction

Recent decades have seen major advances in the treatment of pulmonary arterial hypertension (PAH) [1]; however, PAH remains incurable. Thus, early and accurate risk assessment is recommended to inform treatment decisions and delay disease progression [2]. A goal-oriented treatment approach reduces the mortality of intermediate/high-risk patients and maintains good survival of low-risk patients [3,4].

In clinical practice, subjective risk assessment of PAH patients (i.e., physician gestalt) based on multiple factors (e.g., patient history, laboratory and ambulatory tests, right heart catheterization data, and imaging studies) is common. In addition, several risk assessment tools, validated using data from large PAH registry populations, enable physicians to objectively stratify mortality risk in PAH patients. The Registry to Evaluate Early and Long-Term PAH Disease Management (REVEAL) risk calculator was developed in 2010 to estimate PAH mortality risk based on up to 12 variables [5,6]. This calculator was recently updated (REVEAL 2.0) to include an additional variable and to revise cutoffs for seven variables [7]. Two additional risk assessment methods, the Comparative, Prospective Registry of Newly Initiated Therapies for Pulmonary Hypertension (COMPERA) method [8] and the French Pulmonary Hypertension Registry (FPHR) method [9], incorporate data from up to six variables, using thresholds suggested by the European Society of Cardiology/European Respiratory Society (ESC/ERS) pulmonary hypertension guidelines [10].

PAH patients with World Health Organization (WHO) functional class (FC) II symptoms are often considered to be at low risk for disease progression. By definition, these patients have only mild physical activity limitations and experience discomfort (e.g., dyspnea, fatigue, chest pain) with ordinary physical activities [11]. However, a post hoc analysis of the EARLY (NCT00091715) study, which enrolled only WHO FC II PAH patients, suggests otherwise [12]. In this analysis, depending on the risk assessment method used (COMPERA, FPHR, or REVEAL), 35% to 89% of WHO FC II PAH patients were classified as intermediate or high risk at baseline. Furthermore, at 6 months, 7% to 31% had a worsened risk category, which was directly associated with disease progression and death.

We conducted a retrospective chart analysis to compare the risk status of WHO FC II PAH patients as defined by physicians’ subjective evaluation methods to the application of various objective multiparameter risk assessment tools to better understand how well physicians determine the risk status of PAH patients in diverse clinical practice settings. We also evaluated factors at the physician and patient levels that might explain observed incongruency between physicians’ subjective evaluation and objective multiparameter risk assessment tools. We hypothesized that some WHO FC II PAH patients might be at a moderate-to-high risk and that objective multiparameter risk assessment tools might be better at discriminating risk than physician gestalt.

## Methods

### Physicians

This retrospective study was conducted by surveying pulmonologists or cardiologists with ≥3 years of experience post-training who had treated ≥3 WHO FC II PAH patients in the preceding 12 months. Research was conducted among US centers representing diverse settings, including academic, private, tertiary level, and pulmonary hypertension comprehensive care centers. Eligible physicians participated in a telephone interview and provided all clinical information. A database of information to be captured was developed and used to guide interviews in an open, semi-structured approach. Follow-up emails were sent to all physicians who participated to confirm their consent to have data published. Only physicians consenting (via email) to having their data published were included in the analysis.

### Patient charts

Patient charts were included if patients were WHO FC II per their physicians, had WHO group 1 PAH (idiopathic, heritable, connective tissue disease-associated, or congenital heart disease-associated), and were receiving monotherapy (phosphodiesterase type 5 inhibitor [PDE5i] or endothelin receptor antagonist [ERA]) or dual PAH therapy (PDE5i+ERA). Cases for inclusion were selected by participating physicians based on the criteria above. Since the impetus for this analysis was to identify characteristics of patients who were not being treated with prostacyclin pathway agents, charts were excluded if patients had received a prostacyclin pathway agent within the 3 months before analysis. To define a uniform patient population, charts were also excluded if patients had received riociguat during the prior 3 months since very few patients had such treatment. Pertinent de-identified chart data (three to five patient charts per physician) were provided by physicians. Right heart catheterization metrics were excluded from this evaluation if they were not from the most recent visit. As no personal patient information or patient-identifiable data was used or accessed, patient informed consent and/or Institutional Review Board involvement was not warranted.

### Risk assessment

Each physician provided charts for their eligible patients to a reviewer from an independent analytical services company (Putnam Associates, Boston, MA, USA). Physicians were interviewed (duration: approximately 45–60 minutes) individually by telephone using a semi-structured technique and were asked to provide their own assessment of patient risk (low, intermediate, or high) using their clinical judgment, which may have been informed by risk assessment tools. Subsequently, an independent reviewer performed an objective risk assessment using three methods: the COMPERA method [8], a modified (non-invasive) version of the FPHR method [9], and REVEAL 2.0 [7].

For COMPERA [8] values based on the ESC/ERS guidelines [10] were assigned to WHO FC, 6-minute walk distance (6MWD), brain natriuretic peptide (BNP) or *N*-terminal pro-BNP (NT-proBNP), right atrial pressure, cardiac index, and mixed venous oxygen saturation. A value of 1 was assigned to each variable within the low-risk category, a value of 2 was assigned to each variable within the intermediate-risk category, and a value of 3 was assigned to each variable within the high-risk category. The values were summed and divided by the total number of risk determinants available to yield low (<1.5 points), intermediate (≥1.5 and <2.5 points), and high (≥2.5 points) risk status groups.

For the modified non-invasive FPHR-based method [9], a point was given for each of the following variables that fell within the ESC/ERS guidelines-defined low-risk category [10] for that variable: WHO FC, 6MWD, and BNP or NT-proBNP. The values were summed to yield low- (3 points), intermediate- (2 points), and high-risk (1 point) status groups. No patients scored 0 points because FC II is considered low-risk according to this method, and all patients in the analysis were FC II.

For REVEAL 2.0, risk assessment was performed using the variables and associated thresholds included in the REVEAL 2.0 risk calculator [7]. These include age, sex, PAH etiology, blood pressure, pulse, estimated glomerular filtration rate or renal insufficiency, BNP or NT-proBNP, New York Heart Association classification or WHO FC, 6MWD, and recent hospitalizations. Scores were tallied, and patients were assigned to low- (≤6 points), intermediate- (7 or 8 points), or high-risk (≥9 points) groups. Assessments were conducted using data collected at the most recent clinic visit. The duration of time from diagnostic assessments to most recent clinic visit was variable.

### Factors associated with incongruent risk assessment

Results from multiparameter risk assessments were compared with the physicians’ assessments. Factors that could explain incongruencies between physician gestalt and objective multiparameter risk assessments were explored. Incongruency for this subanalysis was defined as a higher risk classification via ESC/ERS guidelines than the physician’s subjective evaluation. When the physician- and multiparameter-based risk assessments differed, the physician was asked to provide the rationale for their risk assessment at the end of the interview. Potential reasons for incongruency were also assessed objectively, using data from patients’ charts. If the incongruency rationale was unknown or if no incongruency was found, charts for those patients were not included in the subanalysis.

### Statistical analysis

Cross-tabulation with the Pearson’s chi-squared test was used to determine the significance between physician and patient factors and incongruent risk assessment. Continuous data are presented as mean ± standard deviation or median (interquartile range), as appropriate. Categorical data are summarized as discrete values and percentages. All p values are reported as two-tailed; a value of <0.05 was considered statistically significant. Statistical analyses were performed using the statistical package IBM SPSS, version 20 (IBM Corporation; Armonk, NY, USA).

## Results

### Patients

In total, 153 patient charts were obtained from 38 physicians working in self-reported pulmonary hypertension centers (n = 28) or non-pulmonary hypertension centers (n = 10). An additional three physicians did not consent to having their data (comprising 12 patient charts) published and are not included in this analysis. Twenty-nine of the physicians were pulmonologists, and nine were cardiologists. Overall 23 physicians were based in an academic center and 15 were community based. Physicians interviewed saw a median of 60 PAH patients/year (range 8–1200), and approximately 45% of patients had WHO FC II PAH. Patient demographics and disease characteristics are shown in Table 1.

**Table 1:** Patient demographics and clinical characteristics.

Characteristic, n (%)	Patients (N = 153)
**Current age (y)**	
<40	33 (22)
41 to 65	74 (48)
>65	46 (30)
**Sex**	
Female	120 (78)
Male	33 (22)
**Physician-reported activity level** (physician perception, subjectively assessed)	
Inactive	23 (15)
Moderately active	109 (71)
Highly active	21 (14)
**Year of diagnosis**	
2018	23 (15)
2017	30 (20)
2016	29 (19)
2015	19 (12)
2014	10 (7)
Before 2014	42 (28)
**Treatment setting**	
Academic center	95 (62)
Community	58 (38)
**Etiology**	
Idiopathic	87 (57)
Connective tissue disease-associated	49 (32)
Congenital heart disease-associated	15 (10)
Heritable	2 (1)
**Number of comorbidities reported per patient**	
0	16 (11)
1	42 (28)
2	36 (24)
3+	59 (39)
**Comorbidities**	
Obesity	52 (34)
Systemic hypertension	47 (31)
Autoimmune diseases	43 (28)
Depression	35 (23)
Scleroderma	34 (22)
Sleep apnea	24 (16)
Thyroid disease	24 (16)
COPD	18 (12)
Renal insufficiency (eGFR <60 mL/min/1.73 m^2^)	17 (11)
Diabetes	19 (12)
CHD	17 (11)
Liver disease	5 (3)
**PAH medication**	
ERA monotherapy	37 (24)
PDE5i monotherapy	21 (14)
Dual therapy withy ERA + PDE5i	95 (62)

Percentages may not add up to 100% due to rounding.

CHD, congenital heart disease; CTD, connective tissue disease; COPD, chronic obstructive pulmonary disease; eGFR, estimated glomerular filtration rate; ERA, endothelin receptor antagonist; PAH, pulmonary arterial hypertension; PDE5i, phosphodiesterase type 5 inhibitor.

### Risk assessment

Physicians reported that they used risk classification guidelines to help inform their risk assessments in 83% of patients; guidelines were not used in 17% (Table 2). Physicians reported 41% (63/153), 46% (70/153), and 13% (20/153) of patients as being at low, medium, and high risk, respectively, per their gestalt. Rates of concordance between risk assessments made by physician gestalt and the COMPERA method, the modified non-invasive FPHR method, and REVEAL 2.0 were 54%, 43%, and 51%, respectively.

**Table 2:** Physician reports of risk classification guidelines referenced in their risk assessments.

Risk Classification Guidelines	Patient Charts (N = 153)
ESC/ERS Guidelines	82 (54)
ESC/ERS and REVEAL	32 (21)
6WSPH Proceedings	7 (5)
ESC only	3 (2)
REVEAL only	3 (2)
None	26 (17)

Percentages do not add up to 100% due to rounding.

6WSPH, 6th World Symposium on Pulmonary Hypertension; ESC, European Society of Cardiology; ERS, European Respiratory Society; REVEAL, Registry to Evaluate Early and Long-Term PAH Disease Management.

**Fig 1:**
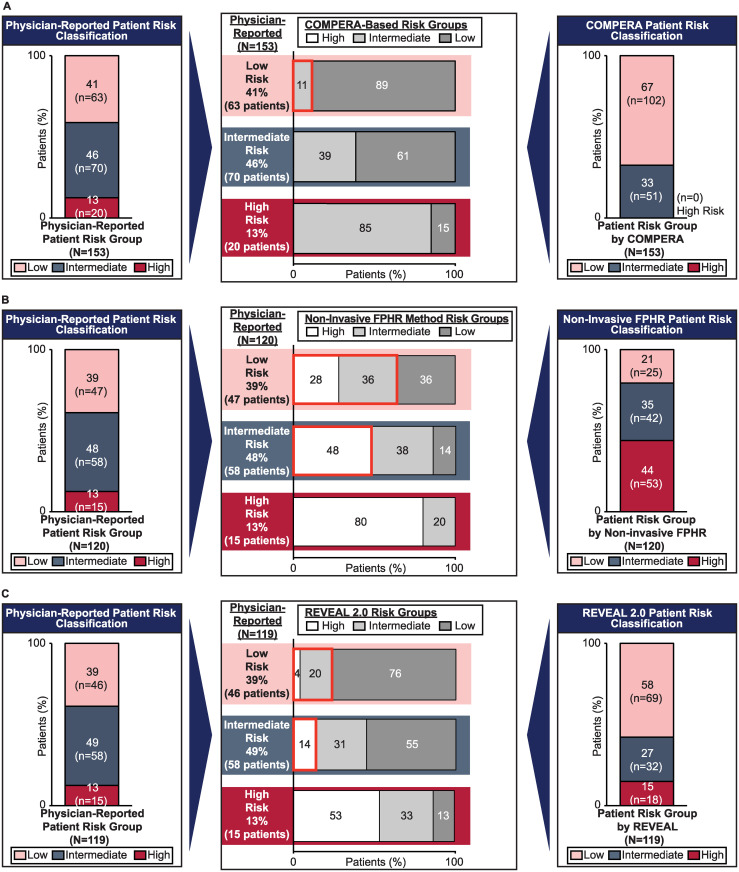
Physician-reported patient risk classification versus A. COMPERA [8], B. non-invasive FPHR [9], and C. REVEAL 2.0 [7]. The COMPERA (A) and FPHR (B) methods use thresholds suggested by ESC/ERS pulmonary hypertension guidelines. The non-invasive FPHR method requires BNP values; 120 patients had available BNP values. The REVEAL 2.0 method (C) requires patients to have complete data for age, sex, etiology, vitals (BP and pulse), eGFR or renal insufficiency, NT-proBNP, NYHA FC, 6MWD, and recent hospitalizations; 119 patients had available data. Red boxes indicate where patients were rated as higher risk by the objective methods than by physicians. 6MWD, 6-minute walking distance; BNP, brain natriuretic peptide; BP, blood pressure; COMPERA, Comparative, Prospective Registry of Newly Initiated Therapies for Pulmonary Hypertension; eGFR, estimated glomerular filtration rate; FPHR, French Pulmonary Hypertension Registry; inter., intermediate; NT-proBNP, *N*-terminal pro-brain natriuretic peptide; NYHA FC, New York Heart Association functional classification; pts, patients; REVEAL, Registry to Evaluate Early and Long-Term PAH Disease Management.

The COMPERA risk assessment classified 67% (102/153) of patients as low and 33% (51/153) as intermediate risk. Comparison of risk assessments (Fig 1A) showed that physician-reported assessment both underestimated and overestimated risk. Eleven percent of the patients classified as low risk by physician gestalt were assessed as being at higher (intermediate) risk by COMPERA. Sixty-one percent of the 70 patients classified as intermediate risk by their physician were classified as low risk by COMPERA, and none of the 20 patients classified as high risk by physicians was classified as high risk by COMPERA.

The modified non-invasive FPHR patient risk was determined in the 120 patients in whom BNP/NT-proBNP values were available; all patients had data available for 6MWD. Using this tool, 21% (25/120), 35% (42/120), and 44% (53/120) of patients were low, intermediate, or high risk, respectively. As with COMPERA, physician-reported assessment both underestimated and overestimated risk compared with the non-invasive FPHR method. Sixty-four percent of the 47 patients classified as low risk per physician gestalt were classified as intermediate (36%) or high (28%) risk by non-invasive FPHR (Fig 1B). Furthermore, of the 58 patients classified as intermediate risk by physician gestalt, 48% were classified as high risk by the non-invasive FPHR method. Fourteen percent of patients reported as intermediate and 20% of patients reported as high risk by physician gestalt were classified as low and intermediate risk, respectively, by non-invasive FPHR.

REVEAL 2.0 examined more parameters and required more complete datasets than the other risk assessment tools employed in the study; consequently, 119 patients had complete data for the REVEAL 2.0 risk assessment. Using REVEAL 2.0, 58% (69/119), 27% (32/119), and 15% (18/119) of patients were low, intermediate, or high risk, respectively. Physician gestalt both underestimated and overestimated risk compared with REVEAL 2.0 (Fig 1C). Of the 46 patients reported as low risk by their physicians, 24% were classified as intermediate (20%) or high (4%) risk using REVEAL 2.0, and 14% of patients reported as intermediate risk by their physicians were classified as high risk by REVEAL 2.0. Fifty-five percent of the 58 patients classified as intermediate risk per physician gestalt were assessed as low risk by REVEAL 2.0, and 47% of patients reported as high risk per physician gestalt were assessed as intermediate (33%) or low (13%) risk by REVEAL 2.0.

### Reasons for incongruent risk assessments

In total, 48 of 153 patient charts were included in the evaluation of physician and patient factors associated with incongruent risk assessment. Thirty charts were excluded as it was not possible to clearly ascertain from the physician the rationale for the incongruency, and 75 were excluded because they were not incongruent. The effect of right heart catheterization metrics on incongruencies could not be evaluated in 79% (38/48) of patient charts because the values were not from the most recent patient visit. Physicians cited patient symptomatic stability (69%) or improvement over time (31%) as the primary reasons for the discordance between their assessment as low vs. intermediate or high risk calculated with objective multiparameter assessment tools.

**Fig 2:**
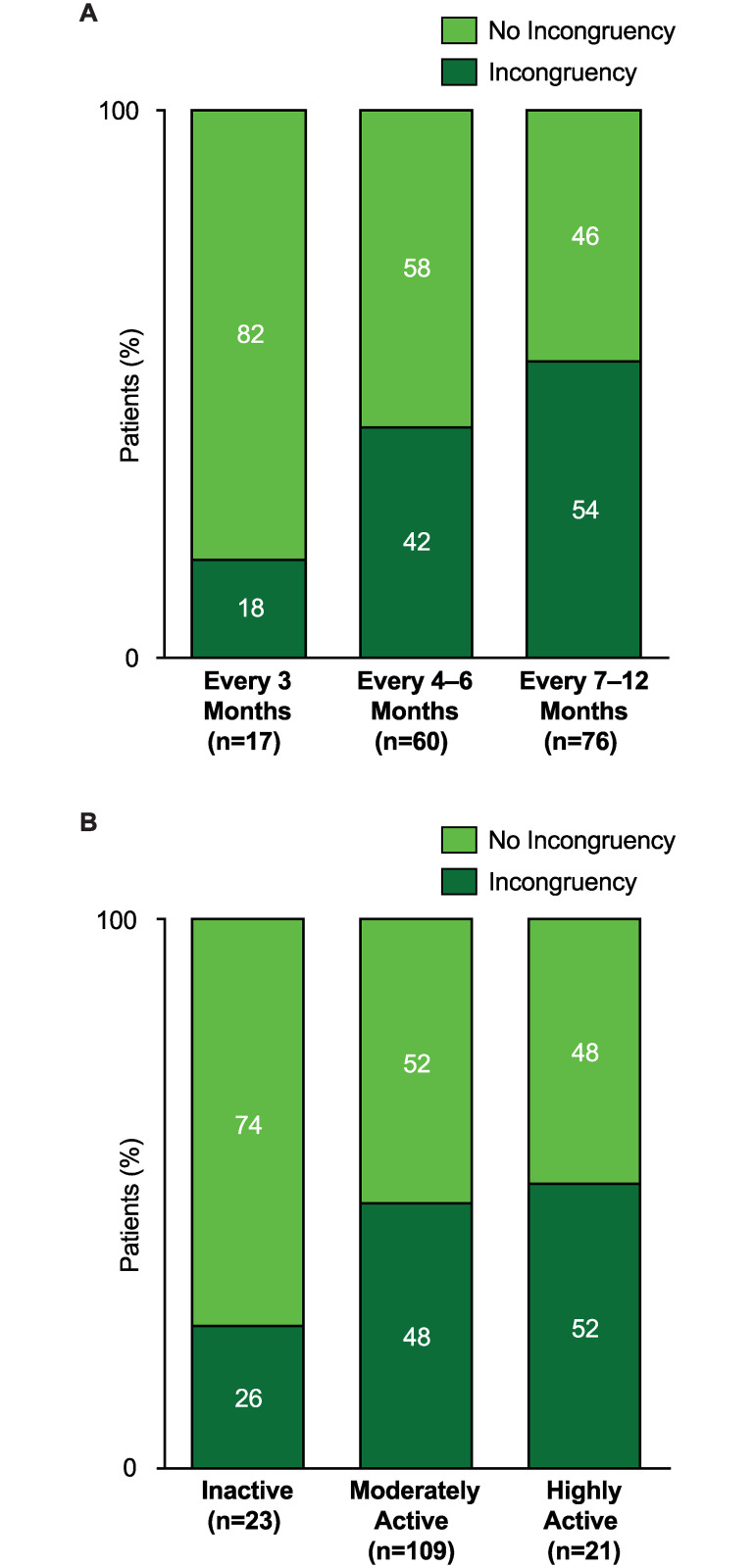
Factors associated with risk assessment incongruency. **(A)** Frequency of echocardiographic monitoring was associated with incongruency such that patients with echocardiography every 3 months were less likely to have incongruency of risk assessment than those with echocardiography every 7 to 12 months (p = 0.01). There was no statistically significant difference in incongruency between patients with echocardiography every 3 months compared to those with echocardiography every 4 to 6 months (p = 0.069). **(B)** The physician-reported activity level was associated with incongruency such that patients with physician-reported high activity levels had more incongruencies than those with physician-reported low activity levels (p = 0.047).

In the evaluation of physician factors associated with incongruent risk assessment, physicians who ordered echocardiography less frequently during follow-up (every 7–12 months vs. every 3 months) were more likely to have incongruencies between their risk assessment and those obtained using multiparametric tools (p = 0.01) (Fig 2A). Other physician factors that were evaluated but not found to correlate with risk incongruency were treatment setting (academic vs. community), physician specialty (pulmonology vs. cardiology), risk classification method used to inform risk assessment (ESC/ERS guidelines, REVEAL, Fifth World Symposium on Pulmonary Hypertension Proceedings [13], none), number of WHO FC II patients, and number of patients treated with oral prostacyclin analogs in the physician’s practice.

Evaluation of patient factors associated with incongruent risk assessments showed a correlation with patient activity, as subjectively assessed by the treating physician: physically active patients were more likely to have incongruent assessments (p = 0.047) (Fig 2B). Patients with comorbidities of systemic hypertension (35% vs. 27% had incongruencies) or autoimmune disease (33% vs. 24% had incongruencies) had numerically more ESC/ERS-based incongruencies than those without these comorbidities. Patients with comorbid obesity were numerically less likely to have incongruencies than non-obese patients (29% vs. 38% had incongruencies). Other patient-associated factors assessed but not significantly associated with incongruencies were year of diagnosis and PAH etiology.

## Discussion

In this retrospective chart review, we found that, depending on the risk assessment method used, 33% to 79% of WHO FC II PAH patients were assessed as intermediate or high risk when using objective multiparameter risk assessment tools. These results suggest that contrary to the concept that patients with WHO FC II symptoms are at low risk, a large proportion of WHO FC II patients are at intermediate or high risk for disease progression and thus may require more intensive therapy.

Our results also show substantial incongruency between physician gestalt and objective risk assessment using three established tools, with concordance rates ranging from 43%–54%. Reliance on clinical judgement for risk assessment (with or without the use of risk tools to help inform risk assessment) resulted in both underestimation and overestimation of risk when compared with assessments made using objective methods. These findings are similar to those published recently from an international survey of physicians’ risk assessment in PAH patients, in which the concordance rate between physician gestalt and objective risk assessment was just 45% [14].

While physicians in our analysis cited a patient’s symptomatic improvement or stability over time as the primary reasons for these incongruencies, an objective analysis found a significant association between less frequent echocardiographic monitoring and underestimation of patient risk by physicians. Our analysis included physicians with ≥3 years of experience post-training; whether more recently trained PAH physicians are more likely to use objective risk assessment tools is undetermined.

The concept of stratifying risk to predict prognosis in PAH patients has evolved relatively recently. The REVEAL risk calculator was developed less than 10 years ago [5], the ESC/ERS risk assessment guidelines were published in 2015 [10], and the COMPERA and FPHR methods were published in 2017 [8,9]. This may explain, in part, why some physicians did not use any objective risk tools to inform their assessments. In contrast, physicians referenced the ESC/ERS risk assessment guidelines when making their risk assessments for more than 50% of patients, suggesting that the guidelines are most often used in a subjective non-systematic way. Physicians were not asked why they did or did not use objective risk assessment tools.

While it is clear discrepancies between physician gestalt and multiparameter objective risk assessments exist, the clinical implications of these in terms of patient outcomes were unclear as was whether any treatment opportunities were missed. Our analysis did show that some physicians underestimated risk vs. objective risk assessment tools and that nearly 70% of those physicians cited patient stability over time per their clinical assessment as the reason for incongruency. This is concerning because studies assessing risk have shown consistently that patients who remain at intermediate or high risk have poorer outcomes compared with patients whose PAH improves to achieve a lower risk profile [4,8,9,15,16]. Our analysis also revealed that patients’ activity levels can mask risk. Moderately or highly active patients were more likely to have incongruencies between physician gestalt and objective risk assessments. Of note, assessments of patient activity were subjective and did not correlate with 6MWD. Therefore, physicians may have overestimated patient activity and, consequently, underestimated risk. Furthermore, physicians who performed echocardiography less frequently were more likely to have patients with incongruencies in risk assessment. According to the ESC/ERS guidelines, echocardiography should be performed every 6 to 12 months, but this interval is “to be adjusted according to patient needs” and increased to every 3 to 6 months after changes in therapy [10]. More frequent assessment may indicate that closer management of patients is being conducted and therefore may lead to early identification of patients that may be declining. Therefore, it would be of interest to establish whether physicians perform echocardiography less frequently in patients they consider to be “active” vs. “inactive,” as that may, at least partially, explain our findings.

Our analysis is limited by the lack of long-term outcomes data, without which we are unable to conclude whether physicians are better or worse than objective algorithms at predicting patient risk. Further follow-up would provide additional insights on choice of risk assessment technique and potential impact on outcomes. Also, due to missing data, not all patients could be assessed by each of the three risk assessment tools. Additionally, we used the modified non-invasive FPHR method, which in the case of all WHO FC II patients comprised only two variables (BNP/NT-proBNP and 6MWD). Moreover, we used this tool to identify patients at any degree of risk (low, intermediate, or high); however, it was originally designed to define patients at low risk for disease progression and mortality. Other limitations include the relatively small sample size of physicians and patient charts examined in a scientific survey setting (which relies on provision of data by physicians and has the potential for recall and/or selection bias), and that only patients receiving either ERA or PDE5i monotherapy, or dual therapy with an ERA and a PDE5i, were eligible. Both of these factors limit the generalizability of our findings. Finally, sample size restricted the use of subset analysis to provide additional granularity (e.g., separate analyses for physicians who stated that formal risk assessment informed their risk evaluation vs. those who said it did not).

This analysis was not designed to compare objective multiparameter risk assessment tools but rather to explore the effect of using these tools in general. However, differences between the calculation methods used and variables included in each risk assessment tool leads to differences in patient risk classification. COMPERA averaging will tend towards median values which means reduced precision and increased variance. REVEAL does not calculate averages, while FHPR uses only two variables as the current analysis is restricted to WHO FC II patients. In conclusion, we found that WHO FC II PAH patients are frequently classified as intermediate or high risk per objective risk assessments and that assessing risk solely by subjective clinical judgment can underestimate or overestimate risk compared with objective risk assessments. The utilization of risk assessment tools could uncover intermediate- and high-risk patients with underestimated risk and complement physician gestalt to better inform treatment decisions. The observed variance between different risk assessment tools and between objective risk assessment and physician gestalt underscores the need for prospective evaluation of risk assessment tools in the real-world setting.

## Supporting information

S1 File Patient database. (XLSM)
